# Supplementary material for: Simultaneous enhancement of strength and conductivity via self-assembled lamellar architecture
Source: Nat Commun. 2024 Feb 29;15:1863. doi: 10.1038/s41467-024-46029-w (PMC10904369; doi:10.1038/s41467-024-46029-w)
Supplement: Supplementary file 1 — Supplementary Information [file 41467_2024_46029_MOESM1_ESM.pdf]

Supplementary Information

Simultaneous enhancement of strength and conductivity via self-assembled lamellar architecture

Tielong Han[1*], Chao Hou[1], Zhi Zhao[1], Zengbao Jiao[2], Yurong Li[1], Shuang Jiang[3], Hao Lu[1], Haibin Wang[1], Xuemei Liu[1], Zuoren Nie[1] & Xiaoyan Song[1*]

[1]*College of Materials Science and Engineering, Key Laboratory of Advanced Functional Materials, Ministry of Education of China, Beijing University of Technology, Beijing 100124, China*
[2]*Department of Mechanical Engineering, The Hong Kong Polytechnic University, Hong Kong, China*
[3]*Key Laboratory of Electromagnetic Processing of Materials (Ministry of Education), School of Material Science and Engineering, Northeastern University, Shenyang 110819, China*

* Corresponding authors. E-mail addresses: tlhan@bjut.edu.cn (Tielong Han); xysong@bjut.edu.cn (Xiaoyan Song)

Supplementary Note 1

Finite element method (FEM) was used to predict the mechanical, electrical and thermal properties of the modeled W-Cu composites with different architectures. According to the distribution characteristics of W phase in the W-Cu composites, generally the architectures can be categorized into five distinct classes. The first class is W-Cu composite reinforced by W particles, where W particles are dispersed in the Cu matrix separately. This type of composite is usually prepared by sintering the Cu-coated W powders. The second class is W-Cu composite reinforced by W skeleton, where W phase forms a connected skeleton, and this type of composite is generally fabricated by melting infiltration. The third class is a hybrid structure, which consists both W particles and W skeleton, and this type of composite is mainly prepared by powders mixing and sintering. The fourth class is W-Cu composite reinforced by W fibers, where W phase is present in the form of fibers. The fifth class is laminated W-Cu composite, where W and Cu layers are distributed alternately.

It is known that refining the structure is beneficial to the strength of the W-Cu composites. Among the five characteristic types of W-Cu composites above, we pay more attention to those whose architectures are feasible to experimentally prepare and refine. Therefore, two of them are selected, i.e., Supplementary Fig. 1a_1 and a_2. Then three representative architectures are evaluated in the following studies: (I) particles reinforced architecture (Supplementary Fig. 1a_1); (II) continuous skeleton architecture (Supplementary Fig. 1a_2); (III) self-assembled laminated (SAL) architecture (Supplementary Fig. 1a_3), which corresponds to the new type of W-Cu composite proposed in this study.

Periodic three-dimensional (3D) representative volume element (RVE) homogenization method is applied for the modeling of W-Cu composites due to its high accuracy [1]. The diameter-to-thickness ratio of W lamellae in the model of SAL W-Cu composite is 15. For all the models, the volume fraction of W is designed to be 51.93%, corresponding to a mass percentage of 70%. The mechanical properties of the W phase and Cu phase are taken from the literature [2,3], with the yield strength and elastic modulus of 900

MPa and 397.8 GPa for W, and 300 MPa and 110 GPa for Cu, respectively (the corresponding true stress-strain curves are shown in Supplementary Fig. 2a). The electrical conductivities of W and Cu are used as 18.8 MS/m (32.41% IACS) and 58.0 MS/m (100% IACS), respectively [4]. The thermal conductivities of W and Cu are used as 175 W/m/K and 398 W/m/K [5], respectively. All the phase interfaces are assumed to be in ideal contact, implying that no interface sliding or cracking would occur with loading, and no additional interface scattering is considered in the simulations of the electrical and thermal conduction processes. Given that the mechanical, electrical and thermal responses strongly depend on the input parameters of material properties and architectures, size effects are not accounted for the simulation [1].

The general linear 3D solid elements C3D10 (10-NODE) are used to mesh the model of all composites. Each 3D structural model of W-Cu composite is divided into more than 100,000 elements in the numerical analysis considering both computational accuracy and time-consuming. The mechanical, electrical and thermal responses along Z-direction are simulated for model I and II, while the mechanical, electrical and thermal responses in both Z-direction (VD) and Y-direction (PD) are investigated for model III due to its anisotropy. For the mechanical simulations, an ABAQUS plugin EasyPBC is used for the imposition of boundary conditions [6], in which the moving region X = Lx (Y = Ly or Z = Lz) constrained with the reference point “RF” could only transfer in Ux (Uy or Uz) DOF (degrees of freedom) along the loading X- (Y- or Z-) direction. The displacement of U = -0.1L is imposed on the reference point “RF”. For the electrical simulations along Z-direction, the voltage V = 0 and V = 1 V are applied separately to the surface at Z = 0 and Z = Lz. Then the electrical resistance along this direction can be calculated. Similarly, the voltage V = 0 and V = 1 V are applied separately to the surface at Y = 0 and Y = Ly when calculating the electrical resistance along Y-direction. For the simulations of the thermal conductivity along Z-direction (Supplementary Fig. 1a_1, a_2 and a_3), a temperature of 293.15 K is applied to the surface at Z = 0, and a heat source with a power of 10^{10} W/m^2 is applied to the surface at Z = Lz. For the simulations of the thermal conductivity of the model in

Supplementary Fig. 1a$_3$ along Y-direction, a temperature of 293.15 K is applied to the surface at Y = 0, and a heat source with a power of 10^3 W/m^2 is applied to the surface at Y = Ly. Thereafter, the thermal conductivities are calculated based on the temperature difference between the two planes (Z = 0 and Z = Lz, Y = 0 and Y = Ly).

The mises stress distribution in the modeled composites, mises stress distribution in the W phase, current density distribution in the modeled composites, and heat flux distribution in the modeled composites are presented in Supplementary Fig. 1b-e, respectively. It can be seen that the W phase always bears more stress than the Cu phase for all the three models (Supplementary Fig. 1b), indicating a stress partitioning effect in the composites. However, as disclosed by Supplementary Fig. 1c and Fig. 2b, the stress distribution and stress level in the W phase is significantly different between the three models, which is closely related to their architectures. A low-stress level in W phase is observed in the composite with a particle-reinforced architecture (model I), the stress values in most areas are lower than the yield strength of W, indicating a low load transfer capacity. That is, the advantage of the high strength of W is not sufficiently exploited in this architecture. In contrast, owing to the unique construction of the SAL architecture (model III), the stress on the W lamellae varies in a range of high values. The stress level on the W skeleton in model II is somewhere in between. Therefore, the W-Cu composite with the SAL architecture shows a potential of a highest compressive yield strength among the three types of composites (Supplementary Fig. 2c).

Furthermore, the electric current density and heat flux distributions within the composites are shown in Supplementary Fig. 1d and e, respectively. It is found that the electric current density and heat flux in the W phase are both significantly lower than those in the Cu phase. Because the electrical resistance of W is much higher than that of Cu, the electrons prefer to flow through Cu phase. It is noted that the distributions of the current density and heat flux are strongly affected by the architecture of the composites. The predicted electrical and thermal conductivities of the composites with different architectures are given in Supplementary Fig. 2d. The SAL W-Cu composite shows significantly higher EC and thermal

conductivity along Y-direction (PD) than the other materials due to the existence of continuous electronic transmission channels. Considering the comprehensive mechanical, electrical, and thermal properties, the SAL W-Cu composite exhibits the best integrated performance.

As the mechanical response of the composites revealed in the FEM simulations is affected by the input parameters of material properties and structure, the actual strength of the prepared W-Cu composites can be different from the simulated yield strength. However, the inputs are the same for the three models, thus the simulation results are comparable. More importantly, the relationship between the designed architecture and the properties of the composite obtained by the simulations is instructive for subsequent studies.

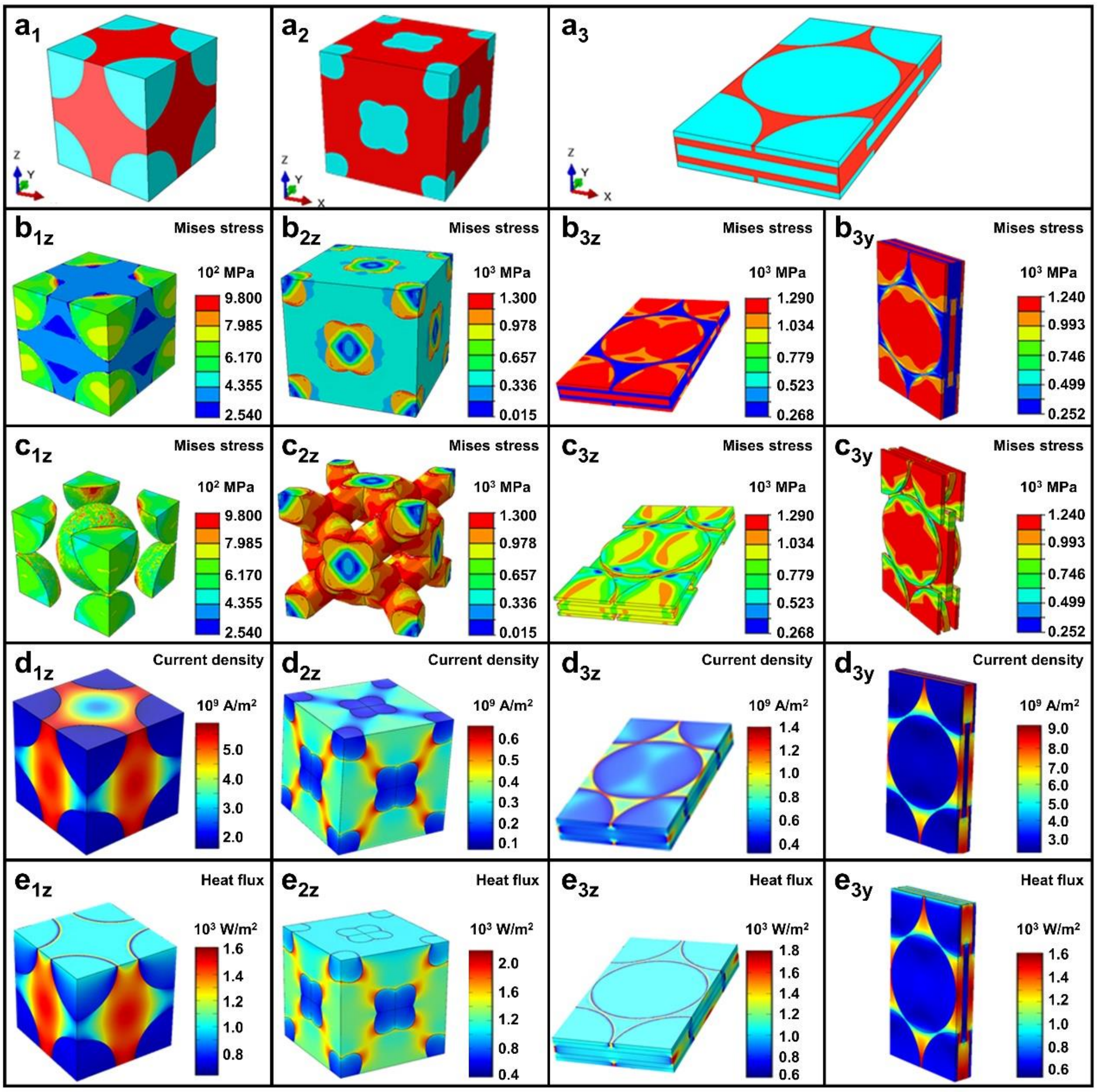

Supplementary Fig. 1 Three-dimensional finite element simulations of the W-30Cu

composites with different architectures. a Periodic representative volume element models of **(a$_1$)** particles reinforced architecture, (**a$_2$**) continuous skeleton architecture and (**a$_3$**) self-assembled laminated architecture, where the red stands for Cu phase and the cyan stands for W phase. **b-e** The corresponding mises stress distribution in the modeled composites (b), mises stress distribution in W phase (c), current density distribution in the modeled composites (d), and heat flux distribution in the modeled composites (e), respectively. The subscript number (1, 2 and 3) indicates the corresponding architecture and the subscript letter (x, y and z) represents the directions in three dimensions.

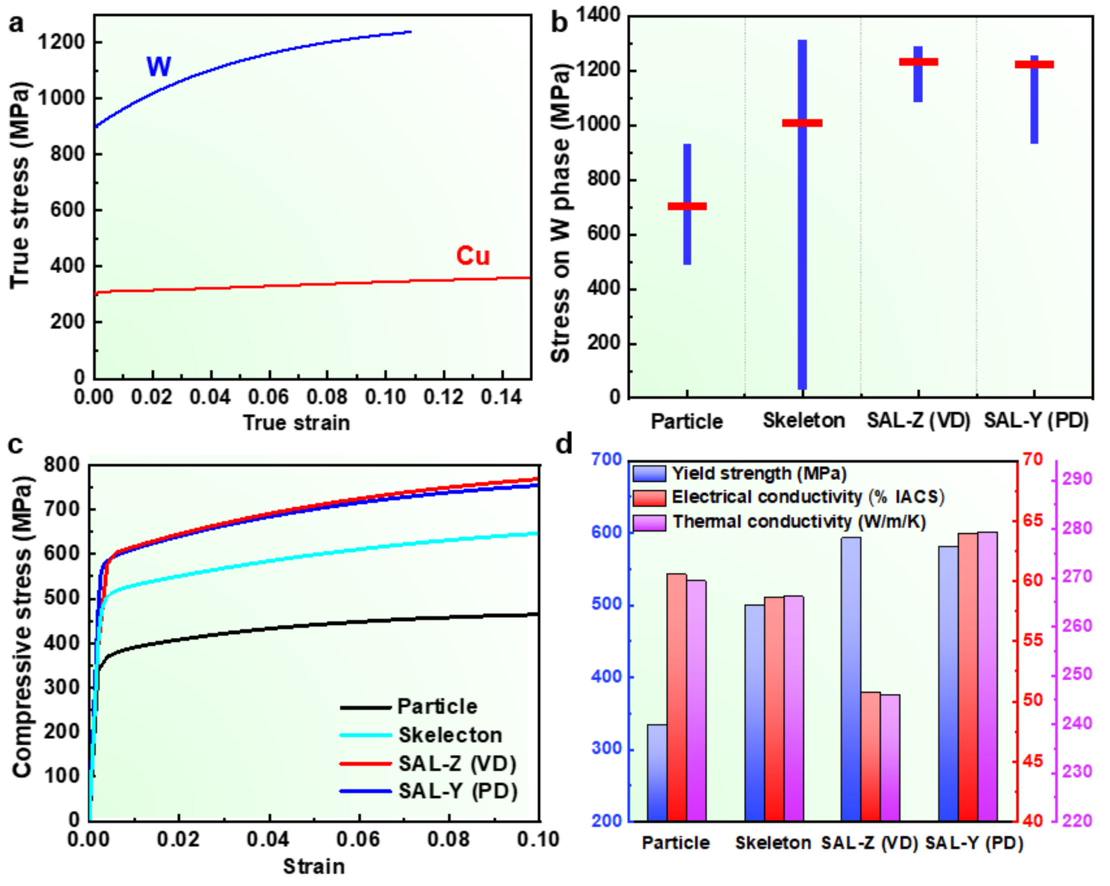

Supplementary Fig. 2 Simulation results of mechanical and conductive behaviors. a True stress-strain curves of Cu and W phases used in the simulation. **b** Stress distribution of the W phase with a compressive strain of 0.1. The red indicates the stress level of most of the elements in the model. **c** Compressive stress-strain curves of W-Cu composites with different architectures. **d** Comparison of the compressive yield strength, electrical conductivity and thermal conductivity of different W-Cu composites.

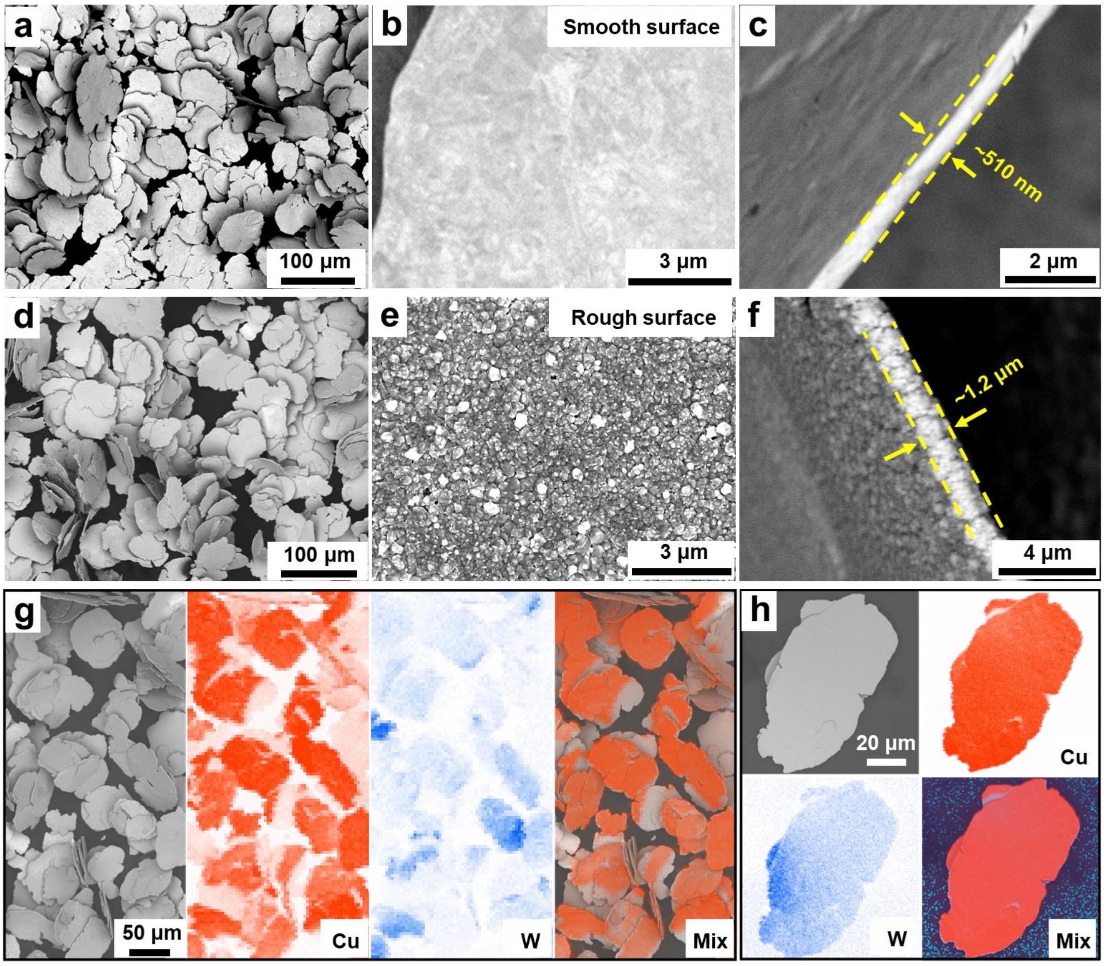

Supplementary Fig. 3 Characterization of the prepared W flakes and Cu-coated W flakes. **a** Low magnification SEM image of W flakes. **b** Surface morphology of the W flake. **c** Side view of the W flake. **d** Low magnification BSE-SEM image of the Cu-coated W flakes. **e** Surface morphology of the Cu-coated W flake. **f** Side view of Cu-coated W flake. **g-h** Energy dispersive X-ray spectrometry (EDS) element mapping of the Cu-coated W flakes, revealing the complete and uniform Cu coating of W flake.

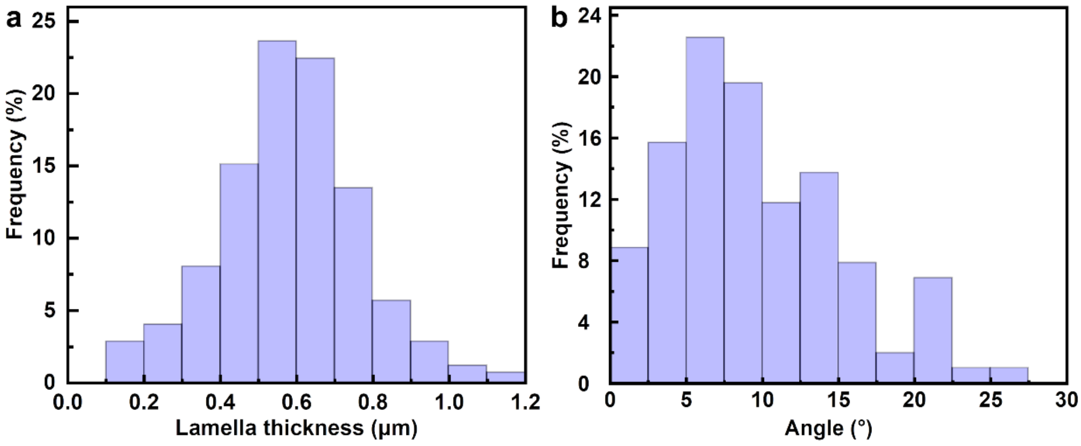

Supplementary Fig. 4 Thickness (a) and bending angle (b) distributions of W lamellae.

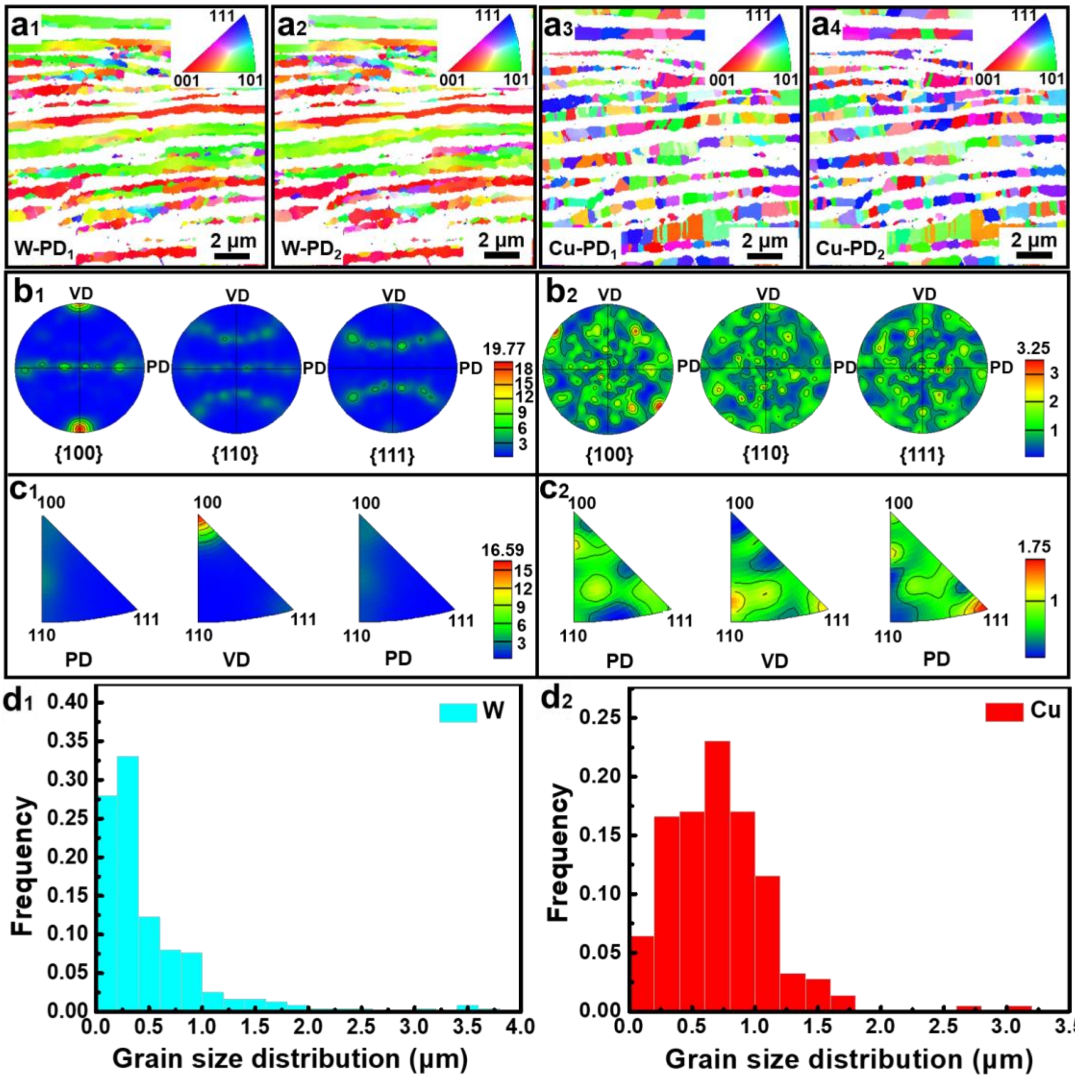

Supplementary Fig. 5 Electron backscatter diffraction analysis of the SAL W-Cu. **a_1-a_4** Crystal orientation maps of W and Cu phases projected on the plane vertical to PD. **b_1-b_2** Pole figures of W and Cu phases. **c_1-c_2** Inverse pole figures of W and Cu phases. **d_1-d_2** Grain size distributions of W and Cu phases.

Supplementary Note 2

Supplementary Fig. 6a shows the X-ray diffraction (XRD) patterns of the SAL W-Cu tested along the in-plane (IP, parallel to PD-PD) and vertical plane (VP, parallel to VD-PD). The standard diffraction patterns of W (PDF#04-0806) and Cu (PDF#04-0836) taken from the JCPDS data are also presented for comparison. The XRD patterns clearly show that no additional peaks are detected except that of W and Cu. The texture coefficient (TC) of the W phase in the SAL W-Cu composite is characterized using the Harris method [7]. The TC is calculated using the following equation [8]:

$$TC = \frac{I_{(hkl)}}{\sum I_{(hkl)}} \cdot \frac{\sum I_{0(hkl)}}{I_{0(hkl)}} \qquad (1)$$

where $I_{(\mathrm{hkl})}$ is the measured relative intensity of the $\{\mathrm{hkl}\}_{\mathrm{W}}$ plane, and $I_{0(hkl)}$ is the standard intensity of the $\{\mathrm{hkl}\}_{\mathrm{W}}$ plane taken from the JCPDS data. A high TC value indicates a strong preferred orientation along a particular crystalline plane of the metal. As shown in Supplementary Fig. 6b, while the TC values of most planes are close to 1, only the TC of $\{200\}_{\mathrm{W}}$ in IP shows an obviously high value, revealing there is a strong texture with $\{100\}_{\mathrm{W}}$ parallel to IP.

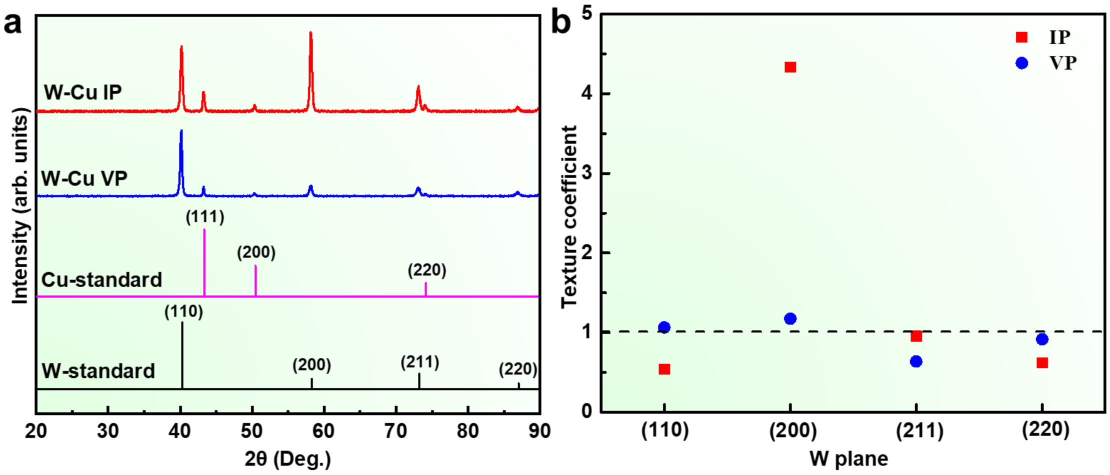

Supplementary Fig. 6 X-ray diffraction analysis of the SAL W-Cu. **a** Typical XRD patterns of SAL W-Cu tested on IP and VP. **b** Texture coefficients calculated for W lamellae on IP and VP by the Harris method.

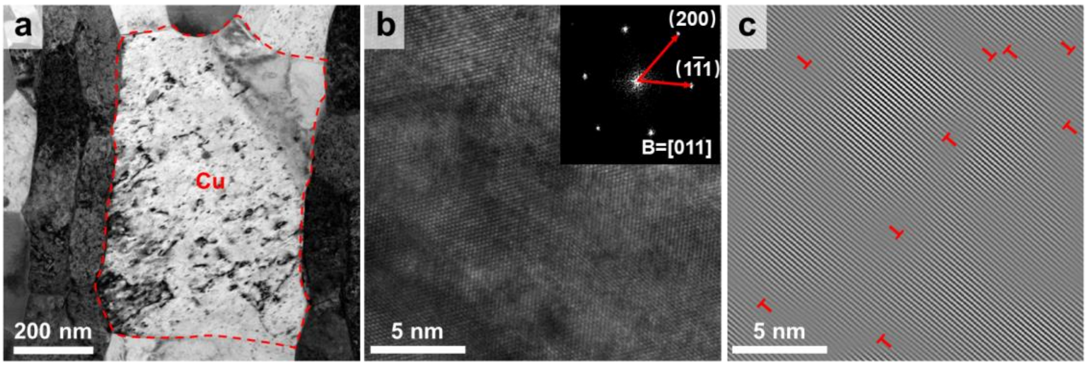

Supplementary Fig. 7 TEM characterization of the Cu phase in the SAL W-Cu composite. a TEM image of the composite where a Cu grain is marked by the red line and dislocations are observed. **b** HRTEM image of the Cu phase. Inset: the corresponding FFT pattern. **c** The inverse FFT pattern of (b), showing the dislocations in Cu phase.

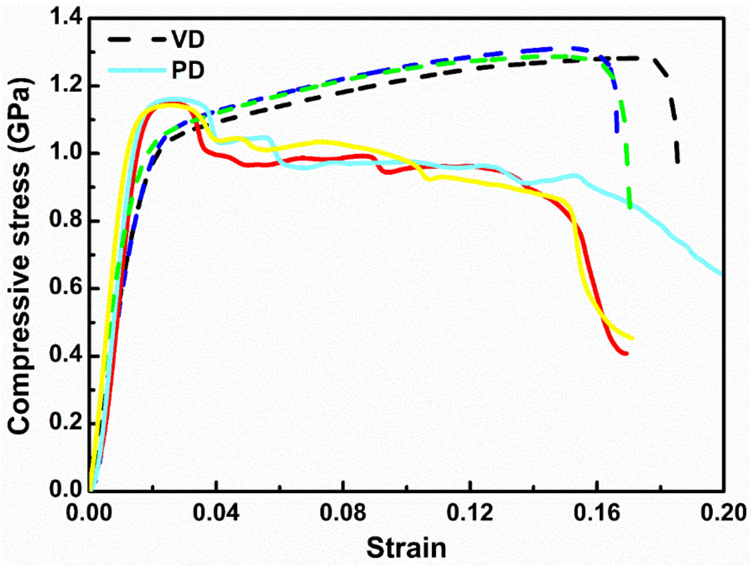

Supplementary Fig. 8 Compressive stress-strain curves of the SAL W-Cu composite.

Supplementary Table 1 Comparison of thermal conductivity of the SAL W-Cu composites with those of the counterparts

Composites	Thermal conductivity ($W\cdot m^{-1}\cdot K^{-1}$)	Preparation methods	Reference
W-30Cu	242	Electroless plating + SPS	This work
W-30Cu	250	Electroplating + Hot pressing	Li. et al. [9]
W-30Cu	241	Pressureless infiltration	Duan. et al. [10]
W-30Cu-4diamond	240	Electroless plating + Microwave sintering	Wei. et al. [11]
W-30Cu	238.3	Metal injection molding	Lee. et al. [12]
W-30Cu	234	Electroless plating + Infiltration	Chen. et al. [13]
	210	Mechanical mixing + Infiltration	
W-30Cu	223.5	Solution combustion synthesis + Sintering	Zhu. et al. [14]
W-30Cu	235.5	Sol-gel procedure + SPS	Guo. et al. [15]
W-30Cu	208	Spray-drying + Sintering	Fan. et al. [16]

Supplementary Note 3

The average stresses of W and Cu phases along VD, denoted as $\bar{\sigma}_{\mathrm{W}}$ and $\bar{\sigma}_{\mathrm{Cu}}$, are calculated by a weighted average of the axial stress in different grains whose {hkl} planes vertical to VD [17,18]:

$$\bar{\sigma}=\frac{\sum_{\mathrm{hkl}}\phi_{\mathrm{hkl}}\sigma_{\mathrm{hkl}}}{\sum_{\mathrm{hkl}}\phi_{\mathrm{hkl}}} \tag{2}$$

$$\phi_{\mathrm{hkl}}=\frac{I_{\mathrm{hkl}}}{\mathrm{K}p_{\mathrm{hkl}}LP_{\theta}A|F_{\mathrm{hkl}}|^{2}\mathrm{e}^{-2M}/v^{2}} \tag{3}$$

$$\sigma_{\mathrm{hkl}}=E_{hkl}\varepsilon_{hkl} \tag{4}$$

where $\bar{\sigma}$ is the average stress in W or Cu, ϕ_{hkl} is the volume fraction of the grains with {hkl} planes, I_{hkl} is the integrated intensity of the {hkl} diffraction peak, K is a scaling factor, p_{hkl} is the multiplicity factor of the {hkl} plane, LP_{θ} is the Lorentz-Polarization factor, A is the absorption factor, F_{hkl} is the structure factor, $\mathrm{e}^{-2\mathrm{M}}$ is the Debye-Waller factor, v is the volume of the unit cell, ε_{hkl} is the lattice strain of the {hkl} plane, and E_{hkl} is the diffraction elastic constant calculated by the ISODEC software, which is shown in Supplementary Table 2.

Supplementary Table 2 The isotropic diffraction elastic constants of W and Cu calculated by the ISODEC software

Phase	W			Cu		
{hkl}	{110}	{200}	{211}	{111}	{200}	{220}
E (GPa)	409.65	409.18	409.65	159.94	102.00	140.05

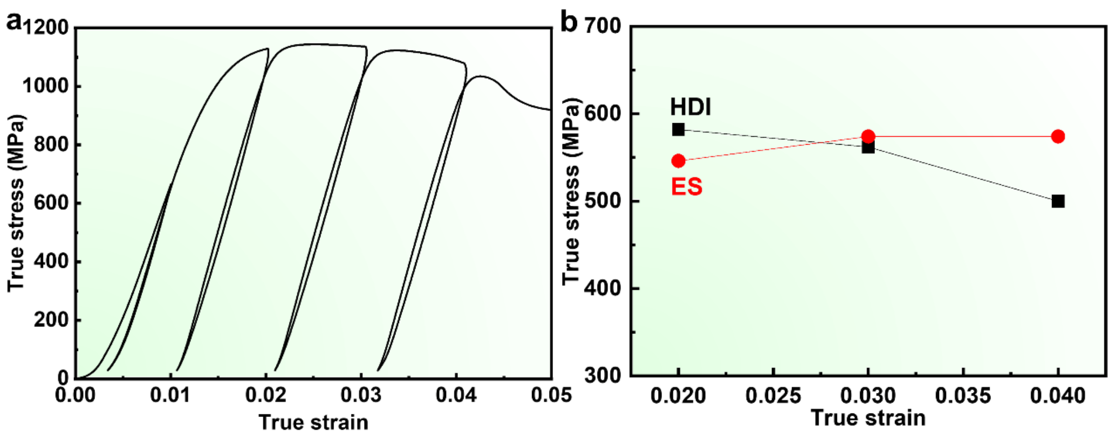

Supplementary Fig. 9 Analysis of the heterogeneous deformation induced stress for the SAL W-Cu compressed along PD. a Loading–unloading–reloading curve of the SAL W-Cu composite along PD. **b** Changes of HDI stress (HDI) and effective stress (ES) with the strain of the SAL W-Cu composite along PD.

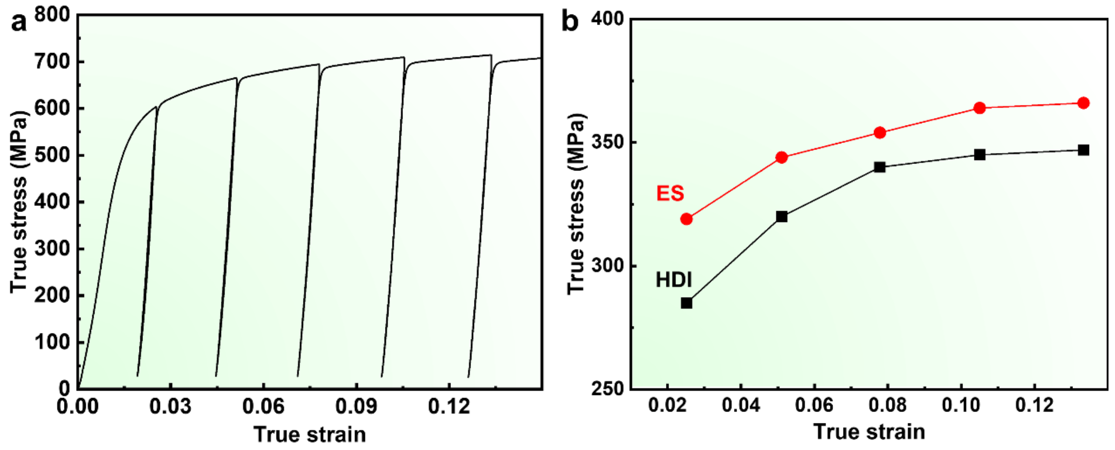

Supplementary Fig. 10 Analysis of the heterogeneous deformation induced stress for the CCG W-Cu. a Loading–unloading–reloading curve of the CCG W-Cu composite. **b** Changes of HDI stress (HDI) and effective stress (ES) with the strain of the CCG W-Cu composite.

Supplementary Note 4

Supplementary Fig. 11a shows the force-displacement curve during the quasi-in-situ compression test, where the force drop is caused by the pause during the photo-taking. As indicated in Supplementary Fig. 11b, we etch some grids on the surface of the sample as markers before the test. The deformation process of the marked regions can also be viewed in the Supplementary Video. It is found that the contrast of the surface before loading is even (Supplementary Fig. 11b). However, after the compression test, lots of bright lines appear near the fracture surface (Supplementary Fig. 11c), which are recognized as microcracks along the interfaces between W and Cu (Supplementary Fig. 11f). Actually, the occurrence of microcracks is at the early stage of plastic deformation of the composite (as seen in Supplementary Video at a displacement of 640 μm). However, further propagation of these microcracks is hindered by the SAL architecture. Hence with the increase of strain, fracture of the composite containing microcracks does not occur, instead, the number of microcracks increases gradually with plastic deformation before failure (Supplementary Fig. 11d and e). As shown in Supplementary Fig. 11d, at a displacement of 720 μm, the crack density is quite high in the microstructure, but the composite can still bear a high load till a displacement of 900 μm (Supplementary Fig. 11a), which indicates a high damage tolerance of the SAL W-Cu composite.

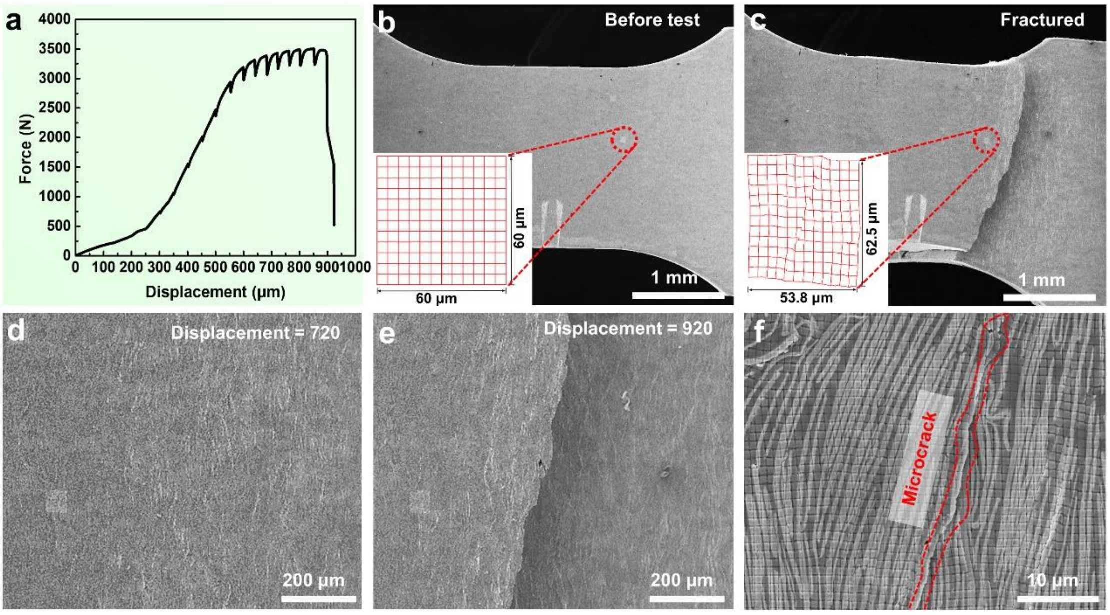

Supplementary Fig. 11 Microcracks in the SAL W-Cu composite during the quasi-

in-situ compression test along VD. a Force-displacement curve in the quasi-in-situ compression test. **b** Image of the sample before loading. Inset: the etched grids before test. **c** Image of the fractured sample after compression test. Inset: the etched grids after test. **d** Microcracks (short curved lines) in the microstructure when the composite is compressed with a displacement of 720 μm. **e** Microcracks in the region close to the fracture surface when the composite is compressed to failure at a displacement of 920 μm. **f** Enlarged view of a microcrack.

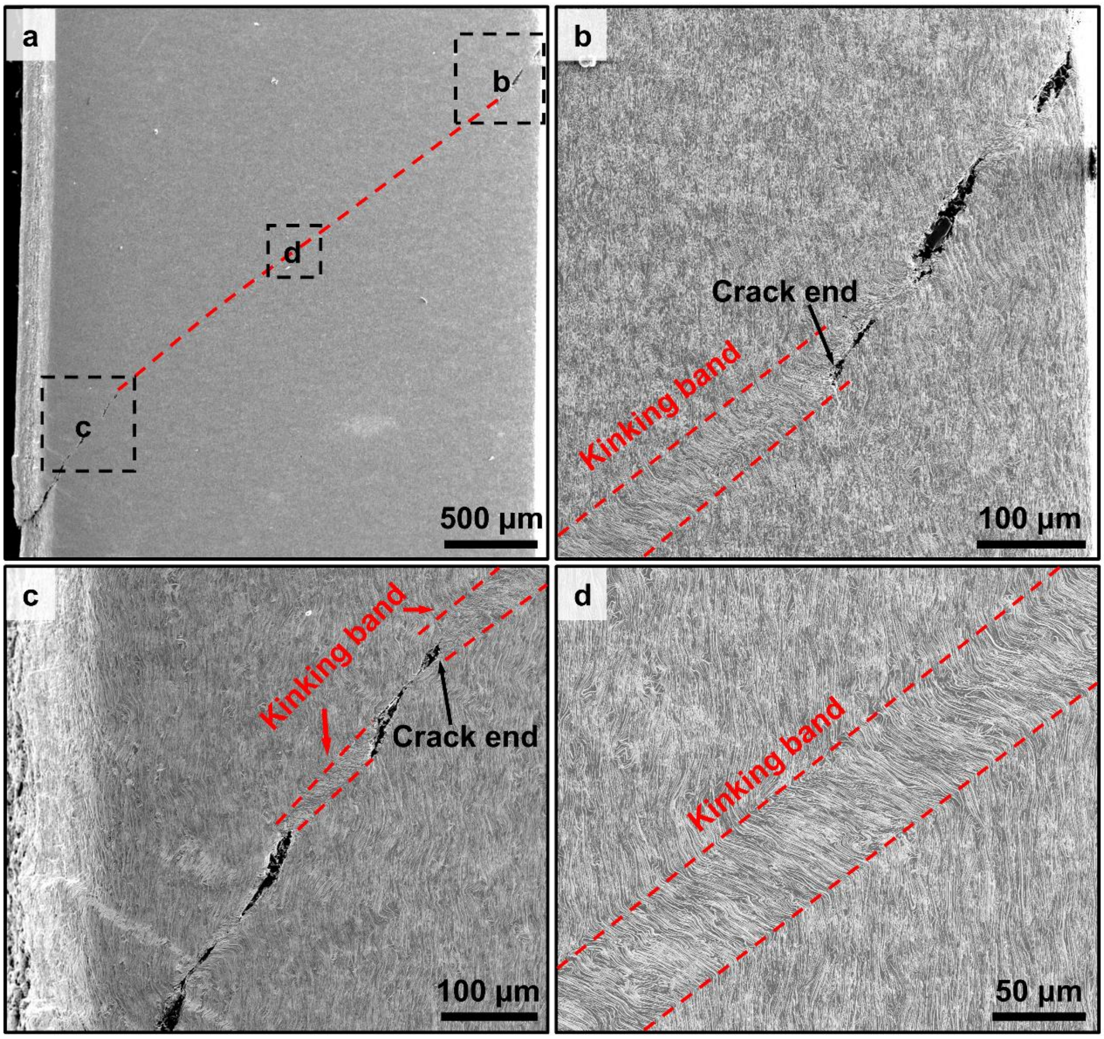

Supplementary Fig. 12 Cross-sectional morphology of the SAL W-Cu composite, sampled after the first drop of the stress when loaded along PD. a The global viewfield. **b-d** Enlargement of local regions in a. Kinking bands and cracks at the ends of the kinking bands are observed.

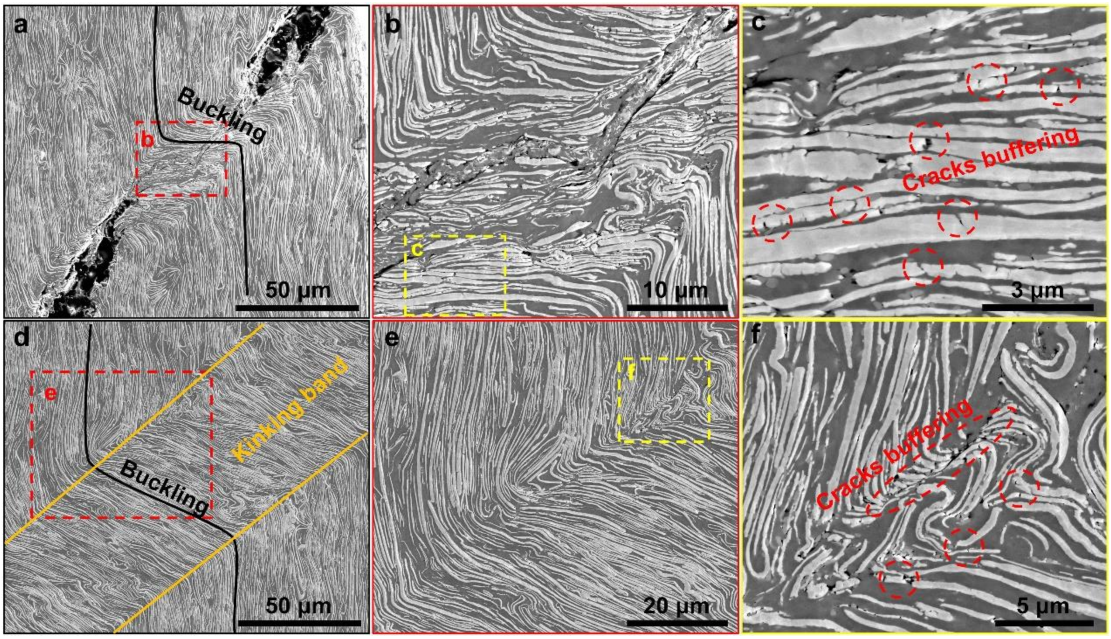

Supplementary Fig. 13 Cross-sectional microstructures of the SAL W-Cu composite after the first drop of the stress when loaded along PD. BSE-SEM images of the regions near (**a-c**) and away from (**d-f**) the crack, where the cracked W lamellae are observed.

Supplementary Note 5

We studied the electrical response of the composite by the FEM simulations based on the real structure of the SAL W-Cu. For comparison, we also analyzed the electrical response of the particles reinforced W-Cu composite. In the simulations, periodic 3D finite element models are used and the volume fraction of W is set as 51.93%, corresponding to a mass percentage of 70% in the W-Cu composite. To take into account the size effect of the interface scattering, a thin interfacial layer with a thickness of 5 nm is introduced with a certain electrical resistance.

As shown in Supplementary Fig. 14a, for the particles reinforced W-Cu model, spherical W particles with a diameter of 14 μm or 600 nm are used in the calculation. The direction that the electrical field is applied is along X-axis. For the SAL W-Cu model, parallel W flakes with a diameter of ~ 55 μm and a thickness of 600 nm are distributed in the Cu matrix randomly (Supplementary Fig. 14b). To understand the effect of W lamella bending on the electrical conductivity (EC) of the SAL W-Cu composite, a modified SAL W-Cu model is set up. In this model, the angle between the W lamella and X-axis is denoted as θ (Supplementary Fig. 14c). For the models of SAL architecture, the directions of the applied electrical field are along X-axis and Z-axis for calculations of EC along PD and VD, respectively.

When the influence of the defects such as voids, dislocations and grain boundaries is not considered, the EC of Cu and W are used as 58 and 18.8 MS/m, respectively. The calculated EC of the particles reinforced W-Cu with two diameters of W particles are all about 60% IACS, which is consistent with the value calculated by the Winer's rule [19]. As shown in Supplementary Fig. 14d, when the interface scattering is considered, the EC of the W particles (14 μm) reinforced W-Cu decreases slightly with the increase of the interfacial resistance, whereas the EC of the W particles (600 nm) reinforced W-Cu decreases seriously. This agrees with the experimental finding that the EC of nanomaterials is generally lower than that of the coarse-grained materials due to the increased fraction of interfaces.

As compared with the particles reinforced W-Cu, the SAL W-Cu exhibits a higher EC along PD and a much lower EC along VD. It is worth noting that the EC of the SAL W-Cu along PD remains almost unchanged with the increase of interfacial resistance. Since the interface is parallel to the direction of electron movement, the interface scattering along PD is greatly diminished in the SAL W-Cu, revealing the advantage of the SAL architecture. However, in the VD direction, the interface is perpendicular to the direction of electron movement, thus EC is severely reduced due to the strong interface scattering.

Supplementary Fig. 14e shows the effect of W lamella bending on EC of the SAL W-Cu, obtained from the simulations with different bending angle of θ. As can be seen, the EC of SAL W-Cu along PD decreases as θ increases, implying that the bending of W lamellae increases the interface scattering. Therefore, the bending angle of the lamella in a laminated composite should be controlled to achieve a high EC. In contrast, the bending of the W lamellae improves EC along VD to some extent.

Further, the influence of the defects on the EC of the composite is considered in the model. As the density of the prepared SAL W-Cu composite is ~ 97%, we assume that the voids are uniformly distributed in the composite, thus the porosities of Cu and W phases are the same as 3%. The simulations show that EC of the phase is reduced by ~ 4.5% due to the porosity. It is reported that the EC of ultrafine-grained metals is about 95% of the theoretical value [20]. Studies by Cetinarslan et al suggested that the reduction of EC due to high-density dislocations is about 2% [21]. Therefore, it is estimated that the EC of Cu and W phases are about 52.62% IACS and 16.72% IACS, respectively. As indicated in Supplementary Fig. 14f, both the reduction of EC in Cu and W lead to a decrease in EC of the composite. Since Cu is a dominant conducting phase when electrons flow along PD, the reduction of EC in Cu would result in a pronounced EC reduction of the composite.

After taking all the factors above into consideration, we obtain two curves showing the evolution of EC as a function of interfacial resistance along PD and VD, respectively (the red curves in Supplementary Fig. 14f).

It is found that the calculated EC of the SAL W-Cu composite, i.e., 56.23% IACS along PD and 33.94% IACS along VD at an electrical resistance of 0.58 MS/m of the interface layer, are close to the experimentally measured values. Therefore, the reduction of EC due to the interface scattering can be determined by the above approach.

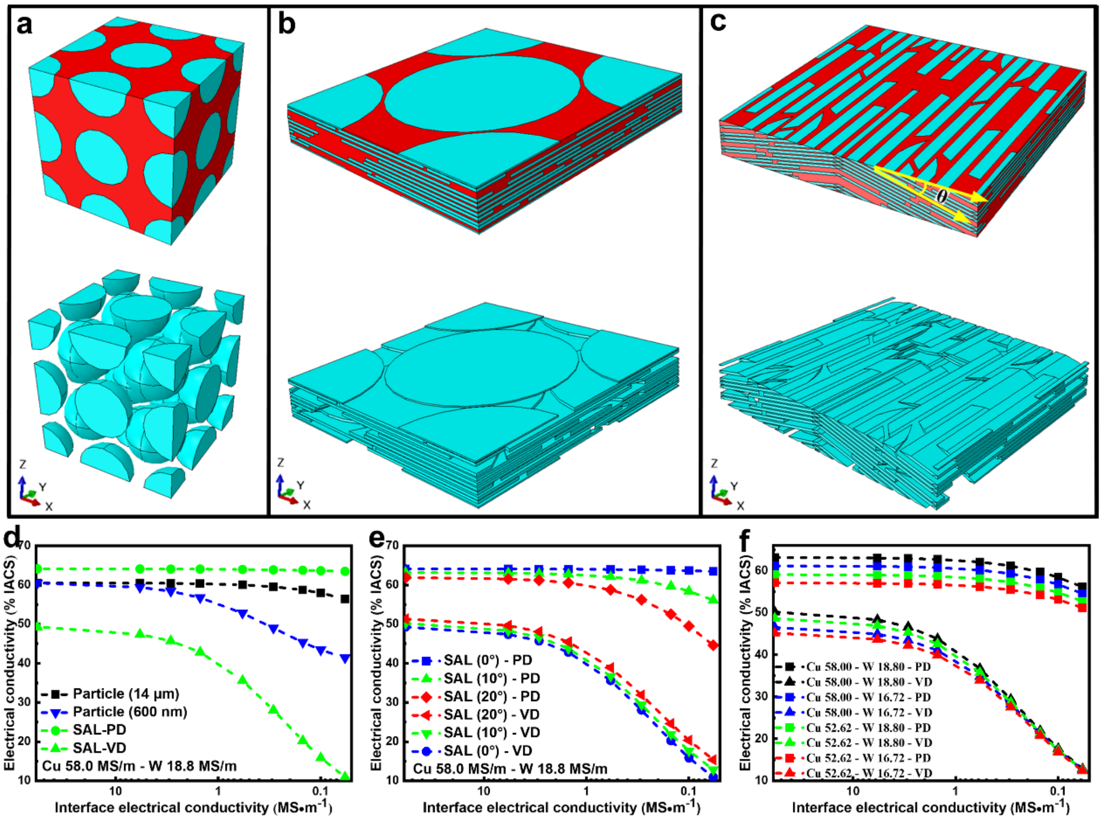

Supplementary Fig. 14 Finite element simulations of electrical conductivity. **a-c** Finite element models of particles reinforced W-Cu composite (a), SAL W-Cu with parallel W lamella (b), and SAL W-Cu with bent W lamella (c). Blue stands for the W phase, and red stands for the Cu phase. **d** Electrical conductivities of modeled composites with different architectures. **e** Effect of the bending angle of W lamella on the electrical conductivity of SAL W-Cu. **f** Effect of defects (voids, dislocations, and grain boundaries) on the electrical conductivity of SAL W-Cu (with a bending angle of W lamella as 10°).

Supplementary Note 6

As indicated in Supplementary Note 1, the thermal conductivities of the SAL W-Cu along PD and VD are 279 and 246 W/m/K, respectively, obtained by simulations under the condition that the effect of defects such as dislocations, voids, grain boundaries and phase interfaces on the thermal conductivity was not considered. The measured thermal conductivities along PD and VD are 242 and 188 W/m/K, respectively. The difference between the measured and the simulated values should be ascribed to the scattering effect of defects on the electrons and phonons and the electron-phonon interactions in the composite [22]. The total thermal conductivity k is mainly composed of the electronic thermal conductivity, k_e, and the phonon thermal conductivity, k_{ph}, i.e., $k = k_e + k_{ph}$ [23]. For metals at room temperature, the k_{ph} is usually much smaller than k_e. It is reported that the k_{ph} of Cu is in a range of 17-22 W/m/K, while the k_{ph} of W is ~ 42 W/m/K [23, 24]. Thus, the contributions of k_{ph} to k are ~5.5% for Cu and ~24.1% for W, respectively. In other words, k_{ph} contributes more to k for W than for Cu. In addition, since both k_e and the electrical conductivity, δ, are closely related to the electrons, their relationship can be described by the Wiedemann-Franz law [24]: $k_e/\delta=LT$, where T is the absolute temperature, L is the corrected Lorenz factor that is material dependent. Therefore, any factors that influence δ would affect k_e proportionally. Moreover, although defects can also affect k_{ph}, the defect scattering has a much stronger effect on the mean free path of electrons than on that of phonons, k_e decreases more than k_{ph}. As a result, k_{ph} has a relatively larger proportion of k with the effect of defects [25]. In other words, although both the k_e and k_{ph} would be affected by the defects, such as dislocations, grain boundaries, and phase interfaces, the effect of defects on k_e is larger than on k_{ph}.

Therefore, if k_{ph} contribution is negligible, one can estimate that the factors influencing δ would affect k proportionally for metals. However, if k_{ph} cannot be neglected, the $k/\delta=(k_e+k_{ph})/\delta$ value would increase. For the conduction along PD in the SAL W-Cu, the measured k/δ (7.45) is similar to the simulated value (7.51), indicating that the contribution of the phonon thermal conductivity is negligible along PD in the SAL W-Cu. In contrast, along VD, the measured k/δ (9.53) is obviously larger than the simulated

value (8.36), implying that the contribution of the phonon thermal conductivity should not be neglected along VD.

Supplementary References

1. Hohe, J. et al. Numerical exploration into the potential of tungsten reinforced CuCrZr matrix composites. *J. Nucl. Mater.* **470**, 13-29 (2016).
2. Xue, P., Xiao, B. L. & Ma, Z. Y. High tensile ductility via enhanced strain hardening in ultrafine-grained Cu. *Mater. Sci. Eng. A* **532**, 106-110 (2012).
3. Lennon, A. M. & Ramesh, K. T. The thermoviscoplastic response of polycrystalline tungsten in compression. *Mater. Sci. Eng. A* **276**, 9-21 (2000).
4. Jones, H. A. A temperature scale for tungsten. *Phys. Rev.* **28**, 202-207 (1926).
5. Deng, N., Zhou, Z., Li, J. & Wu, Y. W–Cu composites with homogenous Cu–network structure prepared by spark plasma sintering using core–shell powders. *Int. J. Refract. Met. Hard Mater.* **82**, 310-316 (2019).
6. Omairey, S. L., Dunning, P. D. & Sriramula, S. Development of an ABAQUS plugin tool for periodic RVE homogenisation. *Eng. Comput. -Germany* **35**, 567-577 (2019).
7. Harris, G. B. Quantitative measurement of preferred orientation in rolled uranium bars. *Philos. Mag.* **43**, 113-123 (1952).
8. Ding, C., Xu, J., Shan, D., Guo, B. & Langdon, T. G. Sustainable fabrication of Cu/Nb composites with continuous laminated structure to achieve ultrahigh strength and excellent electrical conductivity. *Compos. Part B: Eng.* **211**, 108662 (2021).
9. Li, J., Deng, N., Wu, P. & Zhou, Z. Elaborating the Cu-network structured of the W-Cu composites by sintering intermittently electroplated core-shell powders. *J. Alloy. Compd.* **770**, 405-410 (2019).
10. Duan, L., Lin, W., Wang, J. & Yang, G. Thermal properties of W-Cu

composites manufactured by copper infiltration into tungsten fiber matrix. *Inter. J. Refract. Met. Hard Mater*. **46**, 96-100 (2014).

11. Wei, C., Xu, X., Wei, B., Cheng, J. & Chen, P. Effect of diamond surface treatment on microstructure and thermal conductivity of diamond/W-30Cu composites prepared by microwave sintering. *Diam. Relat. Mater*. **104**, 107760 (2020).
12. Lee, S. H., Kwon, S. Y. & Ham, H. J. Thermal conductivity of tungsten-copper composites. *Thermochim. Acta*. **542**, 2-5 (2012).
13. Chen, W. et al. Infiltration sintering of WCu alloys from copper-coated tungsten composite powders for superior mechanical properties and arc-ablation resistance. *J. Alloy. Compd*. **728**, 196-205 (2017).
14. Zhu, X. et al. Preparation and characterization of nanosized W-Cu powders by a novel solution combustion and hydrogen reduction method. *J. Alloy. Compd*. **793**, 352-359 (2019).
15. Guo, Y. et al. Rapid consolidation of ultrafine grained W-30wt.% Cu composites by field assisted sintering from the sol-gel prepared nanopowders. *J. Alloy. Compd*. **724**, 155-162 (2017).
16. Fan, J., Liu, T., Zhu, S. & Han, Y. Synthesis of ultrafine/nanocrystalline W-(30-50)Cu composite powders and microstructure characteristics of the sintered alloys. *Inter. J. Refract. Met. Hard Mater*. **30**, 33-37 (2012).
17. Jiang, S. et al. Micromechanical behavior of multilayered Ti/Nb composites processed by accumulative roll bonding: An in-situ synchrotron X-ray diffraction investigation. *Acta Mater.* **205**, 116546 (2021).
18. Standard practice for X-ray determination of retained austenite in steel with near random crystallographic orientation. *Am. Soc. Test. Mater*. (2013).
19. Han, T. et al. W-Cu composites with excellent comprehensive properties. *Compos. Part B: Eng*. **233**, 109664 (2022).
20. Takata, N., Lee, S. & Tsuji, N. Ultrafine grained copper alloy sheets having both high strength and high electric conductivity. *Mater. Lett.* **63**, 1757-1760 (2009).
21. Çetinarslan, C. S. Effect of cold plastic deformation on electrical

conductivity of various materials. *Mater. Des.* **30**, 671-673 (2009).
22. Dong, L. et al. Thermal conductivity, electrical resistivity, and microstructure of Cu/W multilayered nanofilms. *ACS Appl. Mater. Interfaces* **12**, 8886-8896 (2020).
23. Tong, Z., Li, S., Ruan, X. & Bao, H. Comprehensive first-principles analysis of phonon thermal conductivity and electron-phonon coupling in different metals. *Phys. Rev. B* **100**, 144306 (2019).
24. Stojanovic, N., Maithripala, D. H. S., Berg, J. M. & Holtz, M. Thermal conductivity in metallic nanostructures at high temperature: Electrons, phonons, and the Wiedemann-Franz law. *Phys. Rev. B* **82**, 075418 (2010).
25. Uher, C. Thermal Conductivity of Metals. (Springer, Boston, 2004).
