# Supplementary material for: Simultaneous enhancement of strength and conductivity via self-assembled lamellar architecture
Source: Nat Commun. 2024 Feb 29;15:1863. doi: 10.1038/s41467-024-46029-w (PMC10904369; doi:10.1038/s41467-024-46029-w)
Supplement: Supplementary file 3 — Inventory of Supporting Information [file 41467_2024_46029_MOESM3_ESM.pdf]

Inventory of Supporting Information

File Name: Supplementary Information

Description: Including Supplementary Note 1 to Supplementary Note 6 and Supplementary Fig. 1 to Supplementary Fig. 14.

File Name: Supplementary Movie

Description: Formation and propagation of microcracks during the quasi-in-situ compression test.
